# Supplementary material for: Mutations in PCYT2 disrupt etherlipid biosynthesis and cause a complex hereditary spastic paraplegia
Source: Brain. 2019 Oct 22;142(11):3382–97. doi: 10.1093/brain/awz291 (PMC6821184; doi:10.1093/brain/awz291)
Supplement: awz291_Supplementary_Data [file awz291_supplementary_data.zip › awz291-Suppl_data/Supplementary_Data4.zip!/Supplementary information 3 - fibroblast lipidomics.zip!/Fibroblast lipidomics/Multiple comparison/Heatmap VIP.pdf]

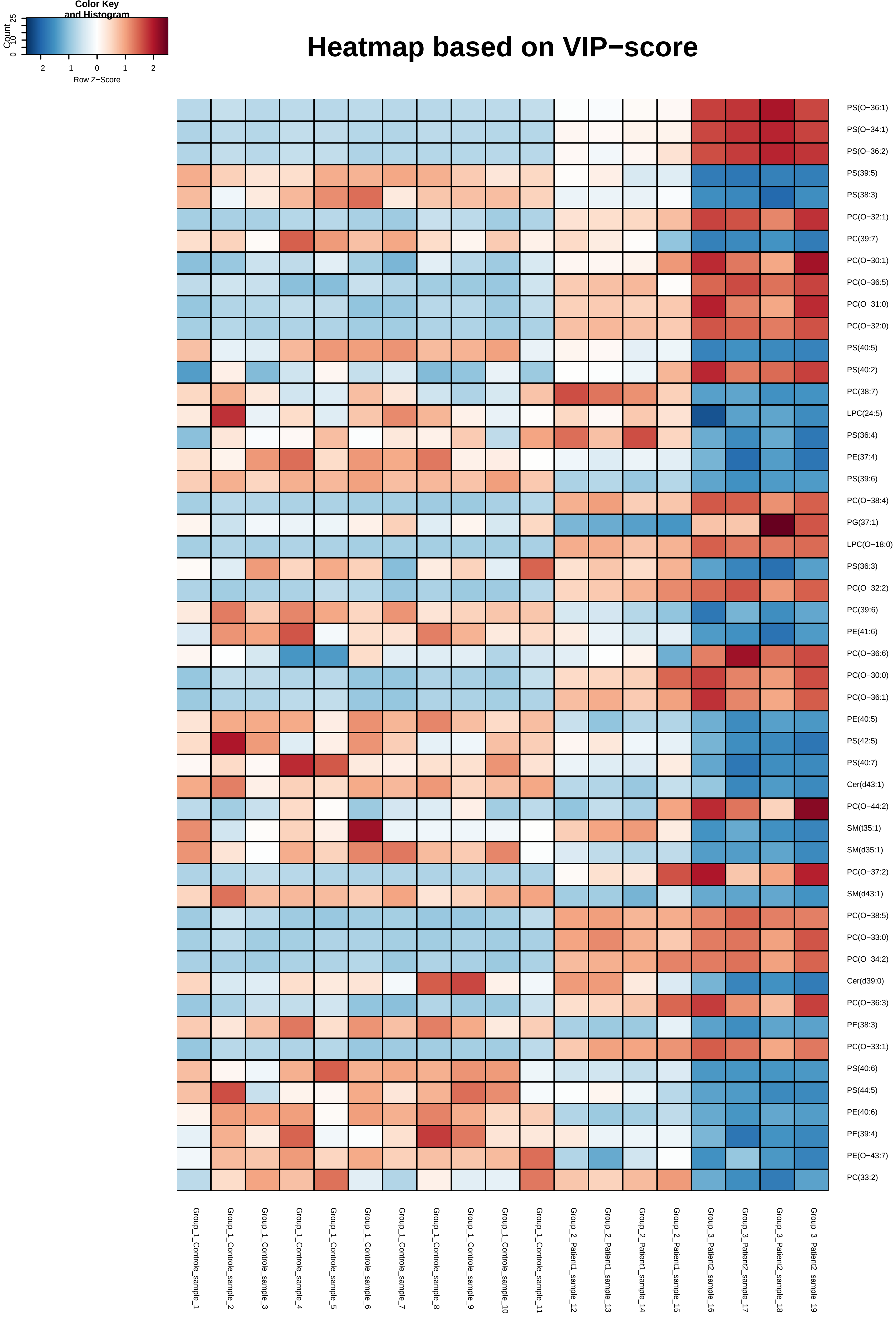
Color Key
and Histogram
Count
0
10
25
−2
−1
0
1
2
Row Z−Score
Heatmap based on VIP−score
PS(O−36:1)
PS(O−34:1)
PS(O−36:2)
PS(39:5)
PS(38:3)
PC(O−32:1)
PC(39:7)
PC(O−30:1)
PC(O−36:5)
PC(O−31:0)
PC(O−32:0)
PS(40:5)
PS(40:2)
PC(38:7)
LPC(24:5)
PS(36:4)
PE(37:4)
PS(39:6)
PC(O−38:4)
PG(37:1)
LPC(O−18:0)
PS(36:3)
PC(O−32:2)
PC(39:6)
PE(41:6)
PC(O−36:6)
PC(O−30:0)
PC(O−36:1)
PE(40:5)
PS(42:5)
PS(40:7)
Cer(d43:1)
PC(O−44:2)
SM(t35:1)
SM(d35:1)
PC(O−37:2)
SM(d43:1)
PC(O−38:5)
PC(O−33:0)
PC(O−34:2)
Cer(d39:0)
PC(O−36:3)
PE(38:3)
PC(O−33:1)
PS(40:6)
PS(44:5)
PE(40:6)
PE(39:4)
PE(O−43:7)
PC(33:2)
Group_1_Controle_sample_1
Group_1_Controle_sample_2
Group_1_Controle_sample_3
Group_1_Controle_sample_4
Group_1_Controle_sample_5
Group_1_Controle_sample_6
Group_1_Controle_sample_7
Group_1_Controle_sample_8
Group_1_Controle_sample_9
Group_1_Controle_sample_10
Group_1_Controle_sample_11
Group_2_Patient1_sample_12
Group_2_Patient1_sample_13
Group_2_Patient1_sample_14
Group_2_Patient1_sample_15
Group_3_Patient2_sample_16
Group_3_Patient2_sample_17
Group_3_Patient2_sample_18
Group_3_Patient2_sample_19
